# Commentary: Clinical skills teaching in UK medical education as exemplified by the BM5 curriculum, Faculty of Medicine, University of Southampton

**DOI:** 10.3205/zma001050

**Published:** 2016-08-15

**Authors:** Anja Timm, Clare Polack

**Affiliations:** 1 University of Southampton, Faculty of Medicine, Academic Unit of Medical Education, Medical Education Development Unit (MEDU), Southampton, UK

## Abstract

This commentary seeks to enable comparisons about clinical skills teaching in Germany and the UK. It outlines the British regulatory environment and its impact on programme design. Through the example of the University of Southampton we show how clinical skills teaching is integrated both vertically and horizontally.

## Zusammenfassung

Dieser Kommentar soll Vergleiche über die Vermittlung klinischer Fertigkeiten in Deutschland und Großbritannien ermöglichen. Er skizziert das regulatorische Umfeld in Großbritannien und seine Auswirkung auf die Programmgestaltung an den medizinischen Fakultäten. Am Beispiel der University of Southampton zeigen wir, wie die Vermittlung klinischer Fertigkeiten sowohl vertikal als auch horizontal integriert ist.

## Introduction

As a European medical school running an undergraduate programme in cooperation with a German healthcare provider, Gesundheit Nordhessen, we have been following the recent developments in German medical education with great interest, especially the recently published recommendations of the Wissenschaftsrat [1] and the establishment of the national catalogue of competencies (http://www.nklm.de citid 28 September 2015). We think it is helpful to explore developments in other countries and we seek to facilitate a comparative perspective with this commentary. 

## UK context

For UK medical schools, all curricula are governed by the national guidance of the General Medical Council (GMC). In “Tomorrow’s Doctors” (2009), the GMC highlights the importance of three domains: The doctor as - ‘a scholar and scientist’, ‘a practitioner’ and ‘a professional’ (http://www.gmc-uk.org/education/undergraduate/undergrad_outcomes.asp cited 29 September 2015). This essentially provides the learning outcomes for all aspects of medical programmes, including non-technical skills (NTS), such as communication and teamwork as well as professional attitudes and behaviours. An appendix outlines 32 practical competencies (http://www.gmc-uk.org/Outcomes_for_graduates_Jul_15.pdf_61408029.pdf cited 29 September 2015). All graduates must achieve both the learning outcomes and the ‘practical procedures’. The overall UK emphasis is on the integration of science/knowledge and its application in clinical practice. Hence most UK medical schools apply an integrated approach to the teaching of practical skills (PS).

Traditional UK undergraduate programmes tend to have the first two years mainly taught at the university, followed by three years mainly in clinical settings. However, a strict division into a pre-clinical and clinical phase has largely been eroded by the ever greater prominence of early patient contact initiatives. This also means that PS and NTS are introduced in year one, initially in quite controlled skills lab settings, then increasingly taught, and practiced in the clinical environment. 

Each medical school designs its curriculum according to its own interpretation of the GMC guidance and local circumstances. However, the GMC quality assures the delivery of each study programme through regular visits and public reporting.

## University of Southampton example

**Figure 1:**
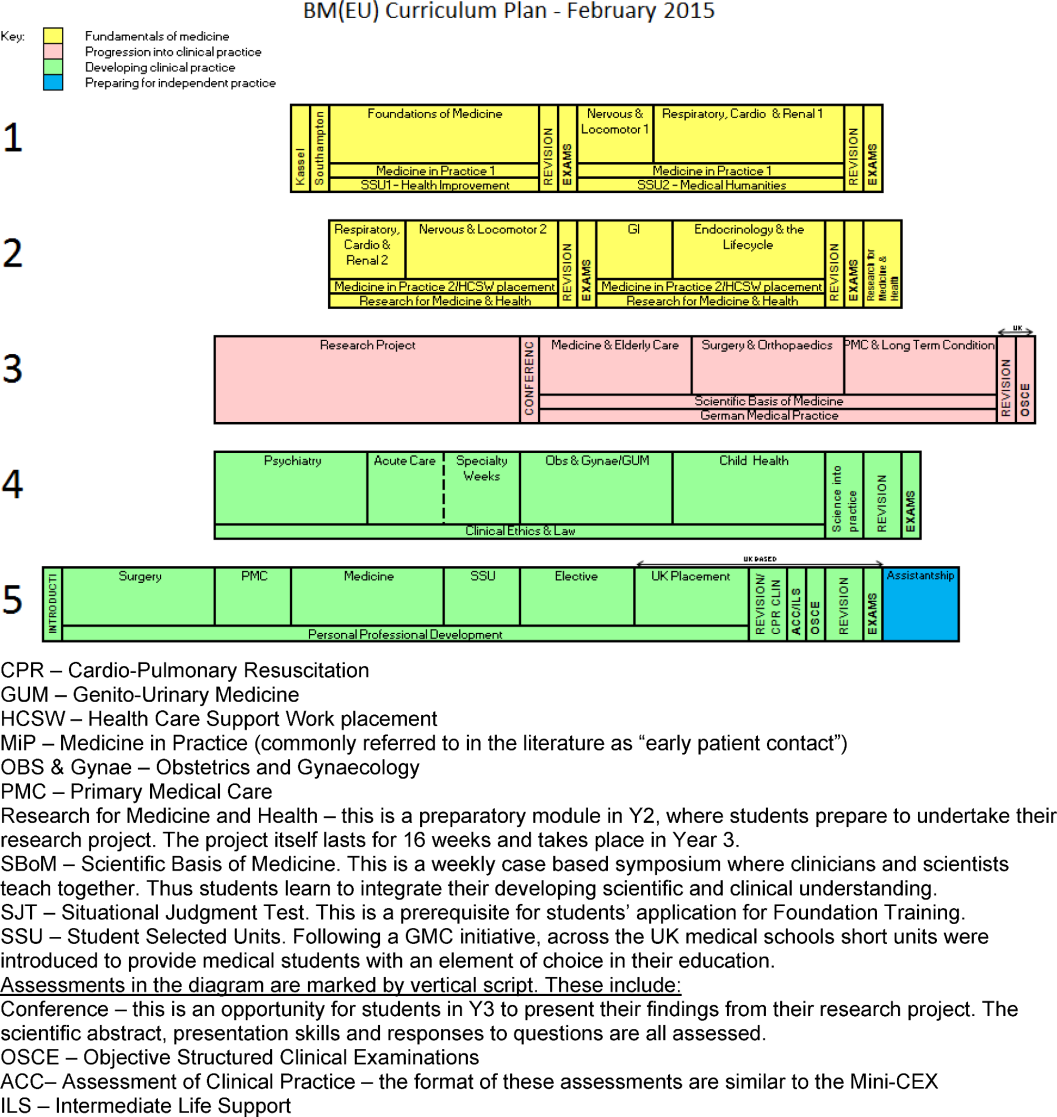
BM5 curriculum map

At Southampton we have an intake of about 290 students annually on five different undergraduate programme streams. The majority undertake the Bachelor of Medicine (BM5) programme, which runs over five years and is divided into four phases, which are demarcated by different colours in the figure below (see Figure 1 (Fig. 1)). 

In each phase PS and NTS are integrated into students’ learning and made relevant by their connection with the science, the patient and the clinical environment.

### 1. Fundamentals of medicine phase (yellow)

- In year 1 PS training is integrated mainly within Medicine in Practice (MiP) but links to the content of the systems-based teaching in the other modules. Whilst students study the Nervous and Locomotor 1 module, they take a history of someone with a musculoskeletal problem. The MiP sessions are delivered to small groups of students by general practitioners in their surgeries. NTS, such as time-management, giving and receiving feedback are also facilitated, highlighted and assessed.
- In year 2 MiP continues as before, although settings alternate between General Practice and hospital. Additionally, all students undertake facilitated health care support work (HCSW). This entails working shifts on a ward where they learn PS and NTS. For example, students are taught basic infection control and they participate in multi-professional teams. During the accompanying facilitated small group tutorials, students are asked to reflect on the nature of teams and how they can work effectively.

#### 2. Progression into clinical practice phase (pink)

At the start of year 3 students concentrate on a research project and learn many PS including accessing and critically appraising evidence, which are vital in both scientific and clinical practice. In parallel to undertaking their research module, they also undertake PS training (e.g. basic life support) as well as separate communication sessions with simulated patients to prepare for full-time clinical placements (for 4 of 5 days a week), which start in semester 2 of year 3.

In the clinical modules of year 3 – Medicine and Elderly Care / Surgery and Orthopaedics / Long Term Conditions and Primary Medical Care – students gradually progress towards meeting their practical competencies; starting from basic familiarisation using models and practicing in-vitro to observation in practice followed by supervised doing. At this stage, students have to record their progress in a log book to show engagement and facilitate discussion with the clinical supervisor at the end of placement. In subsequent years, the requirements on the students increase and their competencies are logged on their e-portfolio (completion is compulsory). 

#### 3. Developing clinical practice phase (green)

Immersion in the clinical environment allows students to learn and consolidate PS and NTS. To evidence competency in the 32 skills mandated by the GMC students must perform each 3 times independently whilst being observed by an experienced clinician. Both PS and NTS are also assessed in OSCE exams, and Assessment of Clinical Competence (ACC). The latter is an observed clinical assessment that includes diagnosis and management and is an adaptation of the postgraduate work-based placed assessment [2]. The ACC allows for a much more realistic in-vivo assessment of a student’s capability, i.e. the “doing” rather than ‘showing’ in Millers pyramid [3]. Continuous supervisor assessment on placements also allow PS and NTS to be assessed in an integrated and holistic manner. Students must pass all four types of assessment (as well as written papers).

#### 4. Preparing for independent practice (blue) phase

This phase occurs after the major examinations, but is assessed and must be passed to allow graduation. In this final phase students put all the PS and NTS into practice by shadowing and assisting a junior doctor in their daily work. The aim is to ensure that all graduates are properly prepared to take on the responsibilities of their first job. In the UK all graduates enter a two year foundation programme which is also quality assured by the GMC and follows a carefully designed curriculum. At the end of the foundation programme junior doctors should possess all the generic PS and NTS to enable them to start their specialist training. 

## Discussion

At Southampton we believe that the knowledge, skills and attitudes required to be a good doctor are equally important. PS and NTS are introduced early in the curriculum and continually revisited in an integrated manner both horizontally within the year and vertically with previous learning [4]. The prominence of these skills in the curriculum and their testing in important assessments leaves students in little doubt how important they are to practicing in the UK.

The list of competencies in Germany seems very comprehensive but it is not yet clear (to us) how this translates in practice to a graduate’s confidence and skills in these areas.

With the free movement of doctors across Europe this is an important area to examine and collaborate on. More shared knowledge could contribute to better induction and supervision of doctors from other European countries and ultimately improve patient safety.

## Competing interests

The authors declare that they have no competing interests.
